# Systemic corticosterone enhances fear memory extinction in rats: Involvement of the infralimbic medial prefrontal cortex GABA_A_ and GABA_B_ receptors

**DOI:** 10.1002/brb3.70043

**Published:** 2024-09-30

**Authors:** Samira Omoumi, Ali Rashidy‐Pour, Seyed Ali Seyedinia, Parnia Tarahomi, Katayoun Sedaghat, Abbas Ali Vafaei, Payman Raise‐Abdullahi

**Affiliations:** ^1^ Research Center of Physiology Semnan University of Medical Sciences Semnan Iran; ^2^ Department of Physiology, School of Medicine Semnan University of Medical Sciences Semnan Iran; ^3^ Student Research Committee, School of Medicine Semnan University of Medical Sciences Semnan Iran

**Keywords:** auditory fear conditioning, fear memory extinction, GABA, glucocorticoids, infralimbic cortex, mPFC

## Abstract

**Purpose:**

The infralimbic (IL) subregion of the medial prefrontal cortex (mPFC) regulates the extinction of conditioned fear memory. Glucocorticoid and gamma‐aminobutyric acid (GABA) receptors are expressed in the mPFC and are also critical in fear extinction. This study investigated the possible interactive effects of the glucocorticoids and GABAergic system in the IL on the regulation of fear extinction.

**Method:**

The rats were trained using an auditory fear conditioning task during which they received three conditioned stimuli (tones, 30 s, 4 kHz, 80 dB), co‐terminated with the three unconditioned stimuli (footshock, 0.8 mA, 1 s). Extinction testing was conducted over 3 days (Ext 1–3). Thirty minutes before the first extinction trial (Ext 1), the rats received bicuculline (BIC, 1 mg/kg/2 mL, intraperitoneal [i.p.]) as a GABA_A_ receptor antagonist or CGP55845 (CGP, 0.1 mg/kg/2 ML, i.p.) as a GABA_B_ receptor antagonist followed by systemic injection of corticosterone (CORT, 3 mg/kg/2 ML, i.p.). Furthermore, separate groups of rats received a bilateral intra‐IL injection of BIC (100 ng/0.3 µL/side) or CGP (10 ng/0.3 µL/side) followed by a systemic injection of CORT (3 mg/kg/2 ML, i.p.) before the first extinction trial (Ext 1). The extracellular signal‐regulated kinase (ERK1) and cAMP response element‐binding (CREB) activity in the IL was examined by Western blot analysis after Ext 1.

**Finding:**

The results indicated that systemic CORT injection facilitated fear extinction and increased the expression of ERK1 but not CREB in the IL. Both systemic and intra‐IL co‐injection of BIC or CGP blocked the effects of CORT on fear extinction and ERK1 expression.

**Conclusion:**

These findings suggest that glucocorticoids and the GABAergic system may modulate fear extinction through the ERK pathway in the IL.

## Graphical abstract

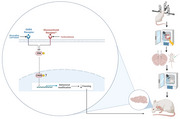

## 1 INTRODUCTION

The formation of fear memories is an adaptive process that enhances survival, but excessive or persistent fear can lead to anxiety disorders like post‐traumatic stress disorder (PTSD) (Abdullahi et al., 2020; Fenster et al., 2018). Fear extinction, the gradual decrease in conditioned fear responses through repeated exposure to feared cues in the absence of aversive outcomes, is a form of inhibitory learning crucial for therapeutic interventions (Meamar et al., 2023). Impairments in fear extinction are implicated in the pathophysiology of PTSD and other anxiety disorders (Meamar et al., 2023; Milad et al., 2009).

Fear extinction involves three phases: acquisition, consolidation, and retrieval (Meamar, Rashidy‐Pour, Rahmani, et al., 2023). During acquisition, new inhibitory associations are formed between the conditioned stimulus and the absence of an aversive outcome. Consolidation is the process of stabilizing and strengthening this newly acquired extinction memory trace. Retrieval refers to the subsequent recall and expression of the extinction memory, leading to reduced fear responses (Giustino & Maren, 2018).

The medial prefrontal cortex (mPFC), particularly the infralimbic (IL) subregion, plays a critical role in the acquisition and consolidation of fear extinction (Dadkhah et al., 2018). Disrupting protein synthesis or neuronal activity in the IL impairs fear extinction learning and memory (Herry et al., 2010). Functional neuroimaging studies in humans have also demonstrated mPFC activation during fear extinction recall (Milad et al., 2007).

Previous studies have indicated the role of several neurotransmitters and hormones, such as gamma‐aminobutyric acid (GABA) (Harris & Westbrook, 1998) and glucocorticoids, which have many receptors in the mPFC, in fear memory reconsolidation and extinction (Dadkhah et al., 2018; Vafaei et al., 2023). Glucocorticoids, being lipid‐soluble, can enter the brain and bind to glucocorticoid receptors (GRs) (de Quervain et al., 2017), affecting several structures in the brain, such as the prefrontal cortex, and modulating different stages of memory (Roozendaal & McGaugh, 1996). Glucocorticoids affect the extinction of conditioned fear (Ali Vafaei et al., 2023; Meamar, Rashidy‐Pour, Vafaei, et al., 2023; Omoumi et al., 2023; Rodrigues et al., 2009). The glucocorticoid synthesis inhibitor, metyrapone, suppresses corticosterone (CORT) synthesis and impairs fear memory extinction (Abdullahi et al., 2020; Barrett & Gonzalez‐Lima, 2004). Similarly, adrenalectomy disrupts the fear extinction (Bohus et al., 1982). Glucocorticoids have rapid effects through nongenomic mechanisms and transmembrane receptors (Khaksari et al., 2007). Nongenomic mechanisms may mediate the impact of CORT on fear memory extinction.

Glucocorticoids interact with neurotransmitter systems in the brain, including GABA, through G‐protein signaling pathways (Di et al., 2009). Dexamethasone has been shown to increase GABA release through rapid nongenomic and nitric oxide‐driven retrograde signaling pathways (Hu et al., 2010). Thus, the effect of glucocorticoids on fear memory extinction may involve interacting with neurotransmitters, particularly the GABAergic system.

The cAMP response element‐binding (CREB) and extracellular signal‐regulated kinase (ERK) proteins in the brain are involved in the process of memory processing, including extinction (Kida et al., 2002). Previous studies have demonstrated that the consolidation of fear extinction relies on the mitogen‐activated protein kinases (MAPK)/ERK signaling pathway and protein synthesis in the prefrontal cortex (Herry et al., 2006; Hugues et al., 2004; Santini et al., 2004). Extinction learning activates ERK and CREB pathways, increasing brain‐derived neurotrophic factor and GluN2B‐containing *N*‐methyl‐d‐aspartate receptors, ultimately resulting in fear memory extinction (Lin et al., 2003).

Despite substantial evidence implicating GABAergic and glucocorticoid systems in fear extinction, the precise mechanisms underlying their interactions in the IL during this process remain unclear. Elucidating these mechanisms could provide valuable insights into the neurobiology of fear regulation and inform the development of novel therapeutic strategies for anxiety disorders. Utilizing the CORT, the ligand for GRs, bicuculline (BIC), the GABA_A_ antagonist, and CGP55845 (CGP), the GABA_B_ antagonist (Jiménez‐Dinamarca et al., 2022; Vafaei et al., 2023), the present study investigates the interactive effects of glucocorticoids and the GABAergic system in the IL on fear extinction and their potential involvement in downstream signaling pathways like ERK1 and CREB.

## 2 MATERIALS AND METHODS

### 2.1 Subjects

The study involved a total of 132 male Wistar rats, aged 10–12 weeks and weighing between 230 and 260 g. The animals were housed in groups of five per cage and maintained under a 12/12 h dark/light cycle at a temperature of 22–24°C, with free access to adequate food and water. All experiments were conducted between 9 am and 3 pm and were carried out in compliance with the Ethics for Laboratory Animals guidelines, as approved by the Medical Ethics Committee at Semnan University of Medical Sciences, Semnan, Iran (approval number: IR.SEMUMS.Rec.1396.243).

### 2.2 Surgical procedure and cannulation

Rats were anesthetized with intraperitoneal (i.p.) injections of ketamine (70 mg/kg) and xylazine (10 mg/kg). Their skulls were immobilized in a stereotactic device, and the coordinates of the IL (2.9 mm Anteroposterior (AP), ±1.0 mm maximum likelihood, 5.0 mm Dorsoventral(DV)) were determined based on the rat brain atlas of Paxinos and Watson (2005). The designated points were punctured with a dental drill, and G23 injection needles (8 mm long, stainless steel) were cannulated bilaterally. The animals were given injections of ketoprofen (2.5 mg/kg, i.p.) and penicillin G (3.5 mg/kg, i.p.) to minimize pain and prevent infection. Following the surgery, the rats were allowed to recover for at least 7 days before undergoing drug injections and behavioral tests (Dadkhah et al., 2018).

### 2.3 Drugs

CORT, BIC, and CGP were obtained from Sigma Co. CORT was dissolved in propylene glycol (PG). BIC and CGP were dissolved in dimethyl sulfoxide (DMSO).

### 2.4 Experimental protocols and drug injections

Rats were randomly divided into 12 groups, with 6 groups for each experiment and *n* = 11 in each group. On Day 1, the animals were habituated to an auditory fear conditioning (AFC) apparatus. On Day 2, the animals were subjected to AFC. Auditory fear extinction was evaluated from Days 3 to 5 (Ext 1–3).

In Experiment 1, rats received BIC (1 mg/kg/2 mL, i.p.) or CGP (0.1 mg/kg/2 mL, i.p.) 30 min before the first extinction trial (Ext 1), followed by systemic injection of CORT (3 mg/kg/2 mL, i.p.) 15 min later. The vehicle (VEH) used for CORT was PG, whereas the VEH for BIC and CGP was DMSO. The VEHs were injected after being diluted with sterile 0.9% saline to reach a concentration of 4% (2 mL/kg, i.p.).

In Experiment 2, the rats received a bilateral intra‐IL injection of BIC (100 ng/0.3 µL/side) or CGP (10 ng/0.3 µL/side) 30 min before Ext 1, followed by a systemic injection of CORT (3 mg/kg/2 mL, i.p.) 15 min later. PG (2 mL/kg, i.p.) and DMSO (0.3 µL/side) were injected as the VEHs. The intra‐IL injections were performed through the cannula embedded in the IL using a Hamilton syringe and infusion pump, with injections administered at a rate of 0.3 µL per 30 s. The needle was kept inside the cannula for an additional 2 min to prevent backflow.

ERK1 and CREB activities were evaluated in the IL. Therefore, after Ext 1, four animals were randomly selected from each group, anesthetized with CO_2_, decapitated, and their brains were removed for Western blot analysis.

### 2.5 Auditory fear conditioning paradigm

The fear conditioning was induced using an AFC apparatus (Borj Sanat Azma Company), which consisted of a wooden outer shield and an inner Plexiglass chamber with a stainless steel bar floor (6 mm in diameter with 12 mm spacing) for administering electric footshocks as the unconditioned stimulus (US) to the animal. The shield roof was equipped with speakers, lamps, and a ventilation system, and a door with a glass valve in the middle formed the front wall of the shield, allowing the animal to be observed during testing. The chamber and shield were connected to a control unit equipped with training interval (TI) recorder software to adjust the test variables and record the data. A trained technician, blinded to the groups, monitored the recorded data every 5 s and marked any instances of freezing on a sheet. Finally, the sum of the marks was multiplied by five to obtain the total freezing time (Omoumi et al., 2023).

#### 2.5.1 Fear conditioning learning protocol

##### 2.5.1.1 Adaptation phase

One day prior to fear conditioning, each animal was introduced to the well‐lit apparatus without any footshock administered, allowing for a 9‐min acclimatization period. During this phase, the device played a total of 12 tones (10 s, 4 kHz, 80 dB, without footshock). After the first six tones, an intra‐TI (ITI) of 3 min elapsed without any audio playback.

##### 2.5.1.2 Training phase

On the second day, the rats were placed in the chamber, and three tones (30 s, 4 kHz, 80 dB) were played from the device. The ITI period was 2 min without any audio playback. During the final seconds of each tone, an electric footshock was applied with an intensity of 0.8 mA for a duration of 1 s.

##### 2.5.1.3 Extinction phase

**FIGURE 1:**
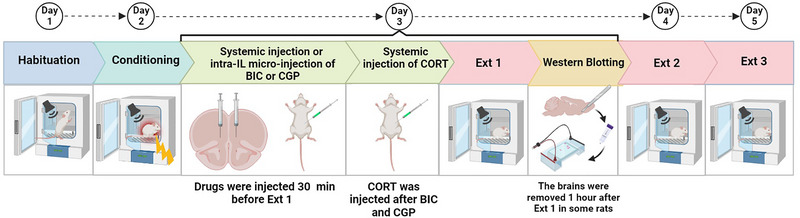
The behavioral training, testing, and drug application timeline is illustrated (see Section 2 for more detail). BIC, bicuculline; CGP, CGP35348; CORT, corticosterone; Ext, extinction trials; IL, infralimbic.

During the extinction phase, the rats were placed in the same chamber where fear conditioning was performed and received 15, 30‐s tones at 2‐min intervals without any footshock. The results were presented as the freezing time observed during each 30‐s tone period. Specifically, the data for the 15 tones in the extinction training were organized into 5 blocks, each representing the average of 3 trials. An experimenter blinded to the experimental groups manually assessed the animals’ freezing behavior. The duration of freezing, defined as the loss of all motor actions except respiratory movements, was calculated during this period. The percentage of freezing was used as an indicator of extinction memory. The extinction trials were conducted 24 h after the training phase on 3 consecutive days (Ext 1–3) (Figure 1).

### 2.6 Western blot analysis

#### 2.6.1 Sample preparation

One hour after the first extinction trial (Ext 1), four rats from each experimental group were randomly selected and deeply anesthetized with CO_2_ inhalation followed by rapid decapitation. The brains were quickly removed and frozen in liquid nitrogen to halt metabolic activity and preserve protein integrity. The frozen brains were stored at −80°C until further processing. The brains were serially sectioned into 180 µm thick coronal slices using a cryostat for tissue extraction. The mPFC region containing the IL area was carefully microdissected from the sections using anatomical landmarks from a rat brain atlas. The extracted IL tissue samples were briefly sonicated in an ice‐cold Radioimmunoprecipitation Assay Buffer (RIPA) lysis buffer (150 mM NaCl, 1% Triton X‐100, 0.5% sodium deoxycholate, 0.1% sodium dodecyl sulfate (SDS), 50 mM Tris pH 8.0) supplemented with protease and phosphatase inhibitor cocktails to disrupt cells and solubilize proteins while preventing degradation. The total protein concentration in each lysate was determined using the Bio‐Rad DC Protein Assay kit (Bio‐Rad) according to the manufacturer's instructions. Samples were adjusted to a final concentration of 5 mg/mL with lysis buffer and 4× Laemmli sample buffer containing β‐mercaptoethanol, boiled for 5 min at 100°C to denature proteins, and stored at −20°C until used for immunoblotting (Tang et al., 2022).

#### 2.6.2 Western blotting

Aliquots containing 30 µg of denatured protein samples were loaded and separated by SDS–polyacrylamide gel electrophoresis using 10% polyacrylamide gels. Following electrophoretic separation, proteins were transferred onto polyvinylidene difluoride membranes (Amersham Biosciences) using a semi‐dry transfer apparatus. The membranes were blocked for 1 h at room temperature in Tris‐buffered saline with 0.1% Tween‐20 (TBST) containing 5% non‐fat dry milk to prevent nonspecific antibody binding. For immunodetection, membranes were incubated overnight at 4°C with gentle agitation in primary antibody solutions prepared in TBST with 5% Bovine Serum Albumin (BSA). Phosphorylated ERK1 (pERK1) was detected using a rabbit monoclonal anti‐phospho‐ERK1 antibody (1:2000; phospho Y204 antibody, ab194770) that recognizes the dually phosphorylated Thr202/Tyr204 activation motif. Total ERK1 protein levels were assessed using a rabbit polyclonal anti‐ERK1 antibody (1:20,000; ab109282) to control for sample loading and protein content. For CREB, a rabbit polyclonal anti‐CREB antibody (1:1000, Millipore) was used to measure total CREB protein levels. After washing off unbound primary antibodies, membranes were incubated for 1.5 h at room temperature with appropriate horseradish peroxidase (HRP)–conjugated secondary antibodies: goat anti‐rabbit IgG (1:5000, Santa Cruz Biotechnology) for pERK1 and goat anti‐rabbit IgG (1:5000, Santa Cruz Biotechnology) for total ERK1 and total CREB. Following secondary antibody incubation and washes, protein bands were visualized by enhanced chemiluminescence using Immobilon Western Chemiluminescent HRP substrate (Millipore) and the UVP AutoChemi imaging system (UVP). To quantify ERK1 levels, the band intensities were densitometrically measured and normalized to their respective total ERK1 levels using ImageJ software (NIH). For CREB, the band intensities were densitometrically measured and represented as total CREB levels using ImageJ software.

### 2.7 Histological examinations

**FIGURE 2:**
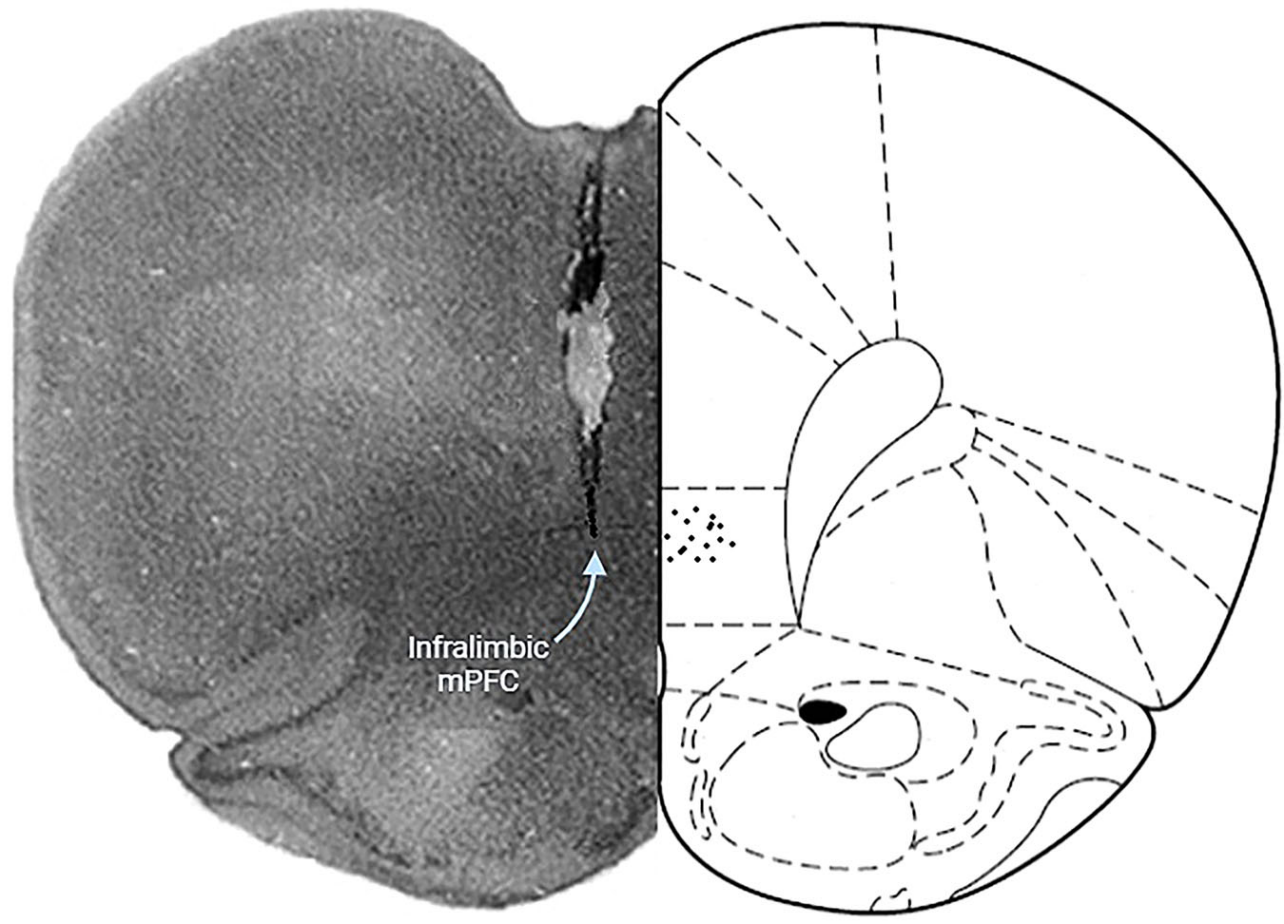
Histological confirmation of the accurate positioning of guide cannulae within the rats. IL, infralimbic; mPFC, medial prefrontal cortex.

After the behavioral experiments, the animals were anesthetized with CO_2_, and their brains were dissected and placed in 10% formalin for 72 h. Cross‐sections of 40‐µm thickness were then prepared, stained with crystal violet, and examined under light microscopy to investigate the cannulation. Results obtained from animals with incorrect cannulation were excluded from the statistical analysis (Figure 2).

### 2.8 Statistical analysis

All data are presented as means ± SEM. Statistical analysis was conducted using two‐way analysis of variance (ANOVA) or three‐way repeated‐measures ANOVA, followed by Tukey's post hoc test when applicable. A significance level of *p* < .05 was considered statistically significant.

## 3 RESULTS

### 3.1 Experiment 1

This experiment investigated the effects of systemic (i.p.) injection of CORT with or without GABA_A_ or GABA_B_ receptor antagonists on fear memory extinction. BIC, or CGP55845, was injected (i.p.) 30 min before Ext 1, followed by an injection of CORT (i.p.) 15 min later.

#### 3.1.1 Conditioning

A three‐way repeated‐measures ANOVA analysis (CORT × GABA antagonist × CS) on the freezing of animals during fear conditioning demonstrated a significant main effect of Conditioned Stimulus (CS) (*F*_2, 72_ = 6090.62, *p* < .0001). The other main effects and interactions were statistically insignificant. Tukey's post hoc test revealed that freezing was significantly higher in the CS 2 and CS 3 compared to the CS 1 in all experimental groups (*p* < .001).

**FIGURE 3:**
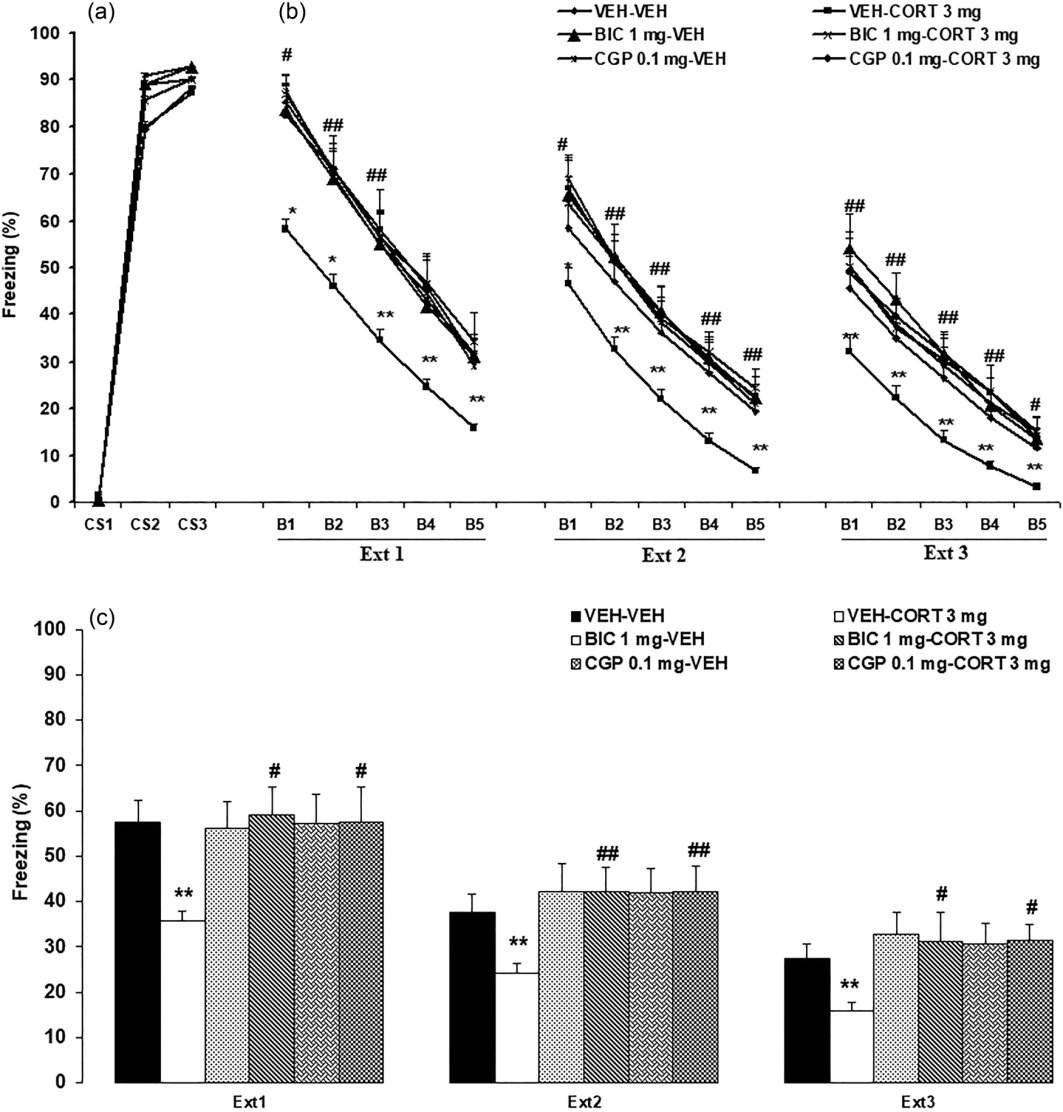
The effects of systemic corticosterone injection on fear extinction after systemic administration of GABA_A_ or GABA_B_ receptor antagonists (bicuculline [BIC] or CGP55845 [CGP], respectively). BIC, CGP, or vehicle was injected (intraperitoneal [i.p.]) 30 min before Ext 1, followed by systemic corticosterone injection 15 min later. (a) Conditioning phase; (b) freezing behavior in blocks 1–5 during each extinction trial; (c) extinction test (average of blocks in three extinction trials). *p < .05 and **p < .01 versus the vehicle (VEH)–VEH (control) group of the previous day; ++*p *< .01 versus the corresponding VEH–VEH (control) groups on each day; ^*p *< .05 versus the VEH–CORT icosterone (CORT) group of the previous day; #*p *< .05 and ##*p *< .01 versus the corresponding VEH–CORT group. The data are presented as mean ± SEM. AFC, auditory fear conditioning; B1‐5, blocks 1–5; Ext 1–3, extinction trials 1–3; GABA, gamma‐aminobutyric acid.

In summary, during the conditioning session, an absence of freezing behavior was observed before the initial presentation of the CS (linked to US). Nevertheless, consistent freezing was exhibited by all six groups during the subsequent second and third CS presentations, highlighting the uniformity among the groups (Figure 3a).

#### 3.1.2 Freezing behavior in blocks 1–5

The extinction data of the experimental groups are shown in Figure 3b.

##### 3.1.2.1 Ext 1

A three‐way repeated‐measures ANOVA analysis (CORT × GABA antagonist × block) on the freezing demonstrated a significant main effect of the block (*F*_4, 144_ = 262.62, *p* < .0001) and a significant interaction between the CORT and GABA antagonist (*F*_2, 36_ = 3.31, *p* = .04). The other main effects and interactions were statistically insignificant. Tukey's post hoc test revealed a significant reduction in freezing response within the VEH–CORT 3 mg compared to the VEH–VEH group (*p* < .05 for B1‐2; *p* < .01 for B3‐5). Furthermore, a significant elevation in freezing was observed within the BIC 1 mg–CORT 3 mg and CGP 0.1–CORT 3 mg compared to the VEH–CORT 3 mg group (*p* < .05 for B1; *p* < .01 for B2‐3).

##### 3.1.2.2 Ext 2

A three‐way repeated‐measures ANOVA analysis (CORT × GABA antagonist × block) on the freezing demonstrated the significant main effects of the GABA antagonist (*F*_2, 36_ = 4.1, *p* = .02) and block (*F*_4, 144_ = 348.78, *p* < .0001). The other main effects and interactions were statistically insignificant. Tukey's post hoc test revealed a significant reduction in freezing response within the VEH–CORT 3 mg compared to the VEH–VEH group (*p* < .05 for B1; *p* < .01 for B2‐5). Furthermore, a significant elevation in freezing was observed within the BIC 1 mg–CORT 3 mg and CGP 0.1–CORT 3 mg compared to the VEH–CORT 3 mg group (*p* < .05 for B1; *p* < .01 for B2‐5).

##### 3.1.2.3 Ext 3

A three‐way repeated‐measures ANOVA analysis (CORT × GABA antagonist × block) on the freezing demonstrated the significant main effects of the GABA antagonist (*F*_2, 36_ = 3.95, *p* = .02) and block (*F*_4, 144_ = 254.31, *p* < .0001). The other main effects and interactions were statistically insignificant. Tukey's post hoc test revealed a significant reduction in freezing response within the VEH–CORT 3 mg compared to the VEH–VEH group (*p* < .01). Furthermore, a significant elevation in freezing was observed within the BIC 1 mg–CORT 3 mg and CGP 0.1–CORT 3 mg compared to the VEH–CORT 3 mg group (*p* < .01 for B1‐4; *p* < .05 for B5).

#### 3.1.3 Extinction test (average of blocks in 3 days)

Figure 3c shows the experimental groups’ Extinction Test data over 3 days (each day represents the average of 5 blocks in the corresponding extinction trial).

A three‐way repeated‐measures ANOVA analysis (CORT × GABA antagonist × extinction) on the freezing demonstrated the significant main effects of the GABA antagonist (*F*_2, 36_ = 5.87, *p* = .006) and extinction (*F*_2, 72_ = 75.92, *p* < .0001) and a significant interaction between the CORT and GABA antagonist (*F*_2, 36_ = 3.34, *p* = .04). The other main effects and interactions were statistically insignificant. Tukey's post hoc test revealed a decline in freezing behavior over the 3 extinction trials (days). Additionally, a significant reduction was found in freezing response within the VEH–CORT 3 mg compared to the VEH–VEH group in Ext 1–3 (*p* < .01). Furthermore, a significant elevation in freezing was observed within the BIC 1 mg–CORT 3 mg and CGP 0.1–CORT 3 mg compared to the VEH–CORT 3 mg group (*p* < .05 for Ext 1 and 3; *p* < .01 for Ext 2).

The rats exhibited increased freezing levels during CSs 2–3 (conditioning day). The freezing was reduced in all groups during five blocks over 3 days. Systemic CORT injection potentiated fear extinction. However, when BIC or CGP55845 (antagonists of GABA_A_ and GABA_B_ receptors, respectively) were administered concurrently, the facilitative impact of CORT on fear extinction was significantly impeded, underscoring the interaction of the glucocorticoid and GABAergic systems in the process of fear extinction.

### 3.2 Experiment 2

This experiment investigated the effects of systemic (i.p.) CORT injection with or without intra‐IL injections of GABA_A_ or GABA_B_ receptor antagonists on fear memory extinction. BIC, or CGP55845, was injected 30 min before Ext 1, followed by an i.p. CORT injection.

#### 3.2.1 Conditioning

A three‐way repeated‐measures ANOVA analysis (CORT × GABA antagonist × CS) on the freezing of animals during fear conditioning demonstrated a significant main effect of CS (*F*_2, 72_ = 8643.5, *p* < .0001). The other main effects and interactions were statistically insignificant. Tukey's post hoc test revealed that freezing was significantly higher in the CSs 2 and 3 compared to the CS 1 in all experimental groups (*p* < .001).

**FIGURE 4:**
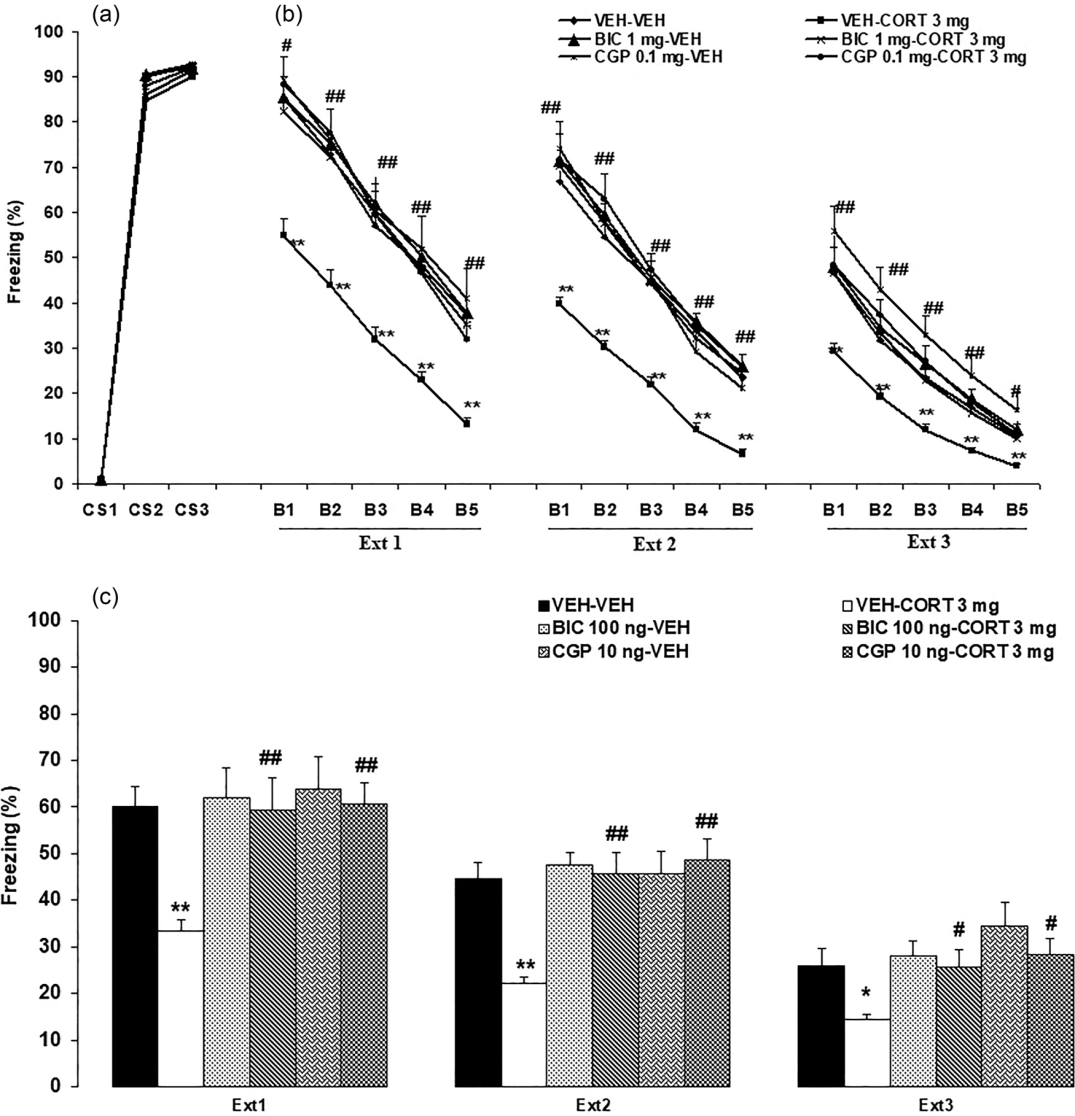
The effects of systemic corticosterone injection on fear extinction after intra‐IL administration of GABA_A_ or GABA_B_ receptor antagonists (bicuculline [BIC] or CGP55845 [CGP], respectively). (a) Conditioning phase; (b) freezing behavior in blocks 1–5 during each extinction trial; (c) extinction test (average of blocks in three extinction trials). *p < .05 and **p < .01 versus the vehicle (VEH)–VEH (control) group of the previous day; ++*p *< .01 versus the corresponding VEH–VEH (control) groups on each day; ^*p *< .05 versus the VEH–CORT icosterone (CORT) group of the previous day; #*p *< .05 and ##*p *< .01 versus the corresponding VEH–CORT group. The data are presented as mean ± SEM. AFC, auditory fear conditioning; B1‐5, blocks 1–5; Ext 1–3, extinction trials 1–3; GABA, gamma‐aminobutyric acid.

In summary, during the conditioning session, an absence of freezing behavior was observed before the initial presentation of the CS (linked to US). Nevertheless, consistent freezing was exhibited by all six groups during the subsequent second and third CS presentations, highlighting the uniformity among the groups (Figure 4a).

#### 3.2.2 Freezing behavior in blocks 1–5

The extinction data of the experimental groups are shown in Figure 4b.

##### 3.2.2.1 Ext 1

A three‐way repeated‐measures ANOVA analysis (CORT × GABA antagonist × block) on the freezing demonstrated the significant main effects of the CORT (*F*_1, 36_ = 7.09, *p* = .01), GABA antagonist (*F*_2, 36_ = 5.96, *p* = .006), and block (*F*_4, 144_ = 263.19, *p* < .0001), and a significant interaction between the CORT and GABA antagonist (*F*_2, 36_ = 3.88, *p* = .02). The other main effects and interactions were statistically insignificant. Tukey's post hoc test revealed a significant reduction in freezing response within the VEH–CORT 3 mg compared to the VEH–VEH group (*p* < 0.01). Furthermore, a significant elevation in freezing was observed within the BIC 100 ng–CORT 3 mg and CGP 10 ng–CORT 3 mg compared to the VEH–CORT 3 mg group (*p* < .05 for B1; *p* < .01 for B2‐5).

##### 3.2.2.2 Ext 2

A three‐way repeated‐measures ANOVA analysis (CORT × GABA antagonist × block) on the freezing demonstrated the significant main effects of the CORT (*F*_1, 36_ = 7.23, *p* = .01), GABA antagonist (*F*_2, 36_ = 11.31, *p* = .0002), and block (*F*_4, 144_ = 324.1, *p* < .0001), and the significant interactions between the CORT and GABA antagonist (*F*_2, 36_ = 8.57, *p* = .0009) and GABA antagonist and block (*F*_8, 144_ = 2.18, *p* = .03). The other main effects and interactions were statistically insignificant. Tukey's post hoc test revealed a significant reduction in freezing response within the VEH–CORT 3 mg compared to the VEH–VEH group (*p* < .05 for B1; *p* < .01 for B2‐5). Furthermore, a significant elevation in freezing was observed within the BIC 100 ng–CORT 3 mg and CGP 10 ng–CORT 3 mg compared to the VEH–CORT 3 mg group (*p* < .01).

##### 3.2.2.3 Ext 3

A three‐way repeated‐measures ANOVA analysis (CORT × GABA antagonist × block) on the freezing demonstrated the significant main effects of the CORT (*F*_1, 36_ = 5.26, *p* = .02), GABA antagonist (*F*_2, 36_ = 5.23, *p* = .01), and block (*F*_4, 144_ = 323.25, *p* < .0001). The other main effects and interactions were statistically insignificant. Tukey's post hoc test revealed a significant reduction in freezing response within the VEH–CORT 3 mg compared to the VEH–VEH group (*p* < .01). Furthermore, a significant elevation in freezing was observed within the BIC 100 ng–CORT 3 mg and CGP 10 ng–CORT 3 mg compared to the VEH–CORT 3 mg group (*p* < .01 for B1‐4; *p* < .05 for B5).

#### 3.2.3 Extinction test (average of blocks in 3 days)

Figure 4c shows the experimental groups’ Extinction Test data over 3 days (each day represents the average of five blocks in the corresponding extinction trial).

A three‐way repeated‐measures ANOVA analysis (CORT × GABA antagonist × extinction) on the freezing demonstrated the significant main effects of the CORT (*F*_1, 36_ = 21.95, *p* < .0001), GABA antagonist (*F*_2, 36_ = 22.99, *p* < .0001), and extinction (*F*_2, 72_ = 82.55, *p* < .0001), and a significant interaction between the CORT and GABA antagonist (*F*_2, 36_ = 11.91, *p* = .0001). The other main effects and interactions were statistically insignificant. Tukey's post hoc test revealed a decline in freezing behavior over the 3 extinction trials (days). Additionally, a significant reduction was found in freezing response within the VEH–CORT 3 mg compared to the VEH–VEH group (*p* < .01 for Ext 1–2; *p* < .05 for Ext 3). Furthermore, there was a significant elevation in freezing observed within the BIC 100 ng–CORT 3 mg and CGP 10 ng–CORT 3 mg compared to the VEH–CORT 3 mg group (*p* < .01 for Ext 1–2; *p* < .05 for Ext 3).

The rats exhibited increased freezing levels during CS 2–3 (conditioning day). The freezing was reduced in all groups during five blocks over 3 days. Systemic CORT injection potentiated fear extinction. However, when intra‐IL injections of BIC or CGP55845 (antagonists of GABA_A_ and GABA_B_ receptors, respectively) were administered concurrently, the facilitative impact of CORT on fear extinction was significantly impeded, underscoring the interaction of the glucocorticoid and GABAergic systems in the process of fear extinction.

### 3.3 ERK1 and CREB analysis

#### 3.3.1 The effect of systemic administration of GABA_A_, or GABA_B_ receptor antagonists, and corticosterone on ERK1 and CREB activities in the IL

**FIGURE 5:**
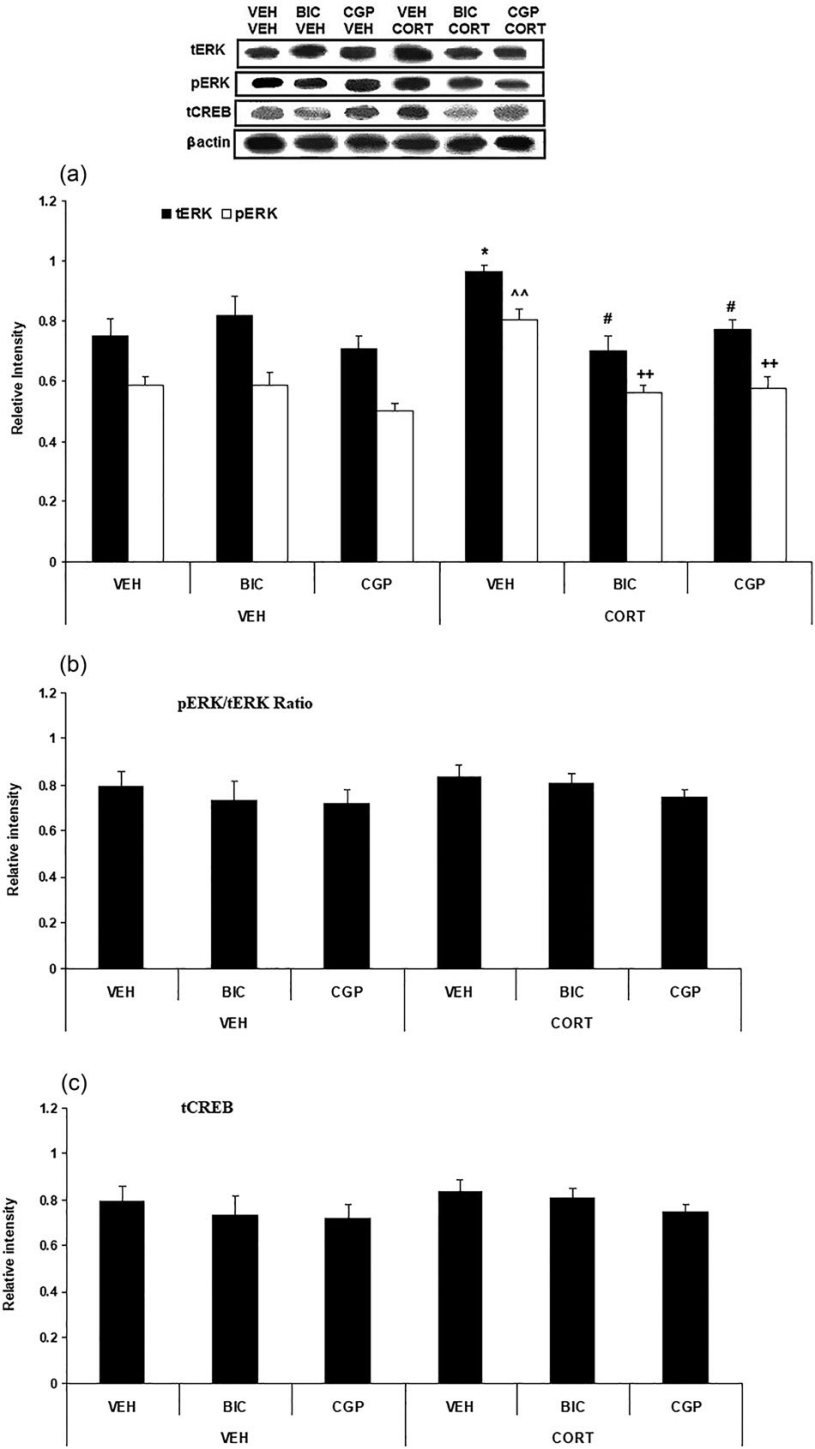
The effects of systemic corticosterone (CORT) injection on the levels of extracellular signal‐regulated kinase (ERK)1 and cAMP response element‐binding (CREB) within the infralimbic (IL) region following the systemic administration of GABA_A_ or GABA_B_ receptor antagonists (bicuculline [BIC] or CGP55845 [CGP], respectively). (a) Total and phosphorylated levels of ERK1; (b) phosphorylated ERK1 (pERK1)/total ERK1 (tERK1) ratio; (c) CREB levels. Data represent the mean ± SEM. *p < .05 versus the tERK1 level in the vehicle (VEH)–VEH (control) group; ^^*p *< .01 versus the pERK1 level in the VEH–VEH (control) group; #*p *< .05 versus the total ERK1 tERK1 level in the VEH–CORT group; ++*p *< .01 versus the pERK1 level in the VEH–CORT group. The data are presented as mean ± SEM. GABA, gamma‐aminobutyric acid.

This experiment investigated the effects of systemic administration of GABA_A_, or GABA_B_ receptor antagonists, and CORT on ERK1 and CREB levels in the IL. The data from the ERK1 and CREB analyses are shown in Figure 5a–c.

A two‐way ANOVA analysis (CORT × GABA antagonist) on tERK1 relative intensity indicated no significant effects of CORT (*F*_1, 18_ = 1.86, *p* = .18), a significant effect of GABA antagonist (*F*_2, 18_ = 3.48, *p* = .05) and a significant interaction between two factors (*F*_2, 18_ = 5.95, *p* = .01). Tukey's post hoc test revealed a significant elevation of tERK1 within the VEH–CORT 3 mg compared to the VEH–VEH group (*p* < .05). Furthermore, there was a significant reduction of tERK1 within the BIC 1 mg–CORT 3 mg and CGP 0.01 mg–CORT 3 mg compared to the VEH–CORT 3 mg group (*p* < .05) (Figure 5a).

A two‐way ANOVA analysis (CORT × GABA antagonist) on pERK1 relative intensity indicated significant effects of CORT (*F*_1, 18_ = 10.99, *p* = .003) and GABA antagonist (*F*_2, 18_ = 12.63, *p* = .0004) and significant interaction between two factors (*F*_2, 18_ = 6.71, *p* = .006). Tukey's post hoc test revealed a significant elevation of pERK1 within the VEH–CORT 3 mg compared to the VEH–VEH group (*p* < .01). Furthermore, there was a significant reduction of pERK1 within the BIC 1 mg–CORT 3 mg and CGP 0.01 mg–CORT 3 mg compared to the VEH–CORT 3 mg group (*p* < .01) (Figure 5a).

A two‐way repeated‐measures ANOVA analysis (CORT × GABA antagonist) on pERK1/tERK1 ratio indicated no significant effects of CORT (*F*_1, 18_ = 1.07, *p* = .31) and GABA antagonist (*F*_2, 18_ = 1.06, *p* = .36), and no significant interaction between two factors (*F*_2, 18_ = 0.11, *p* = .89) (Figure 5b).

A two‐way ANOVA analysis (CORT × GABA antagonist) on tCREB relative intensity indicated no significant effects of CORT (*F*_1, 18_ = 2.93, *p* = .10) and GABA antagonist (*F*_2, 18_ = 2.08, *p* = .15) and no significant interaction between two factors (*F*_2, 18_ = 0.44, *p* = .648) (Figure 5c).

#### 3.3.2 The effects of intra‐IL injections of GABA_A_, or GABA_B_ receptor antagonists and systemic administration of corticosterone on ERK1 and CREB activities in the IL

**FIGURE 6:**
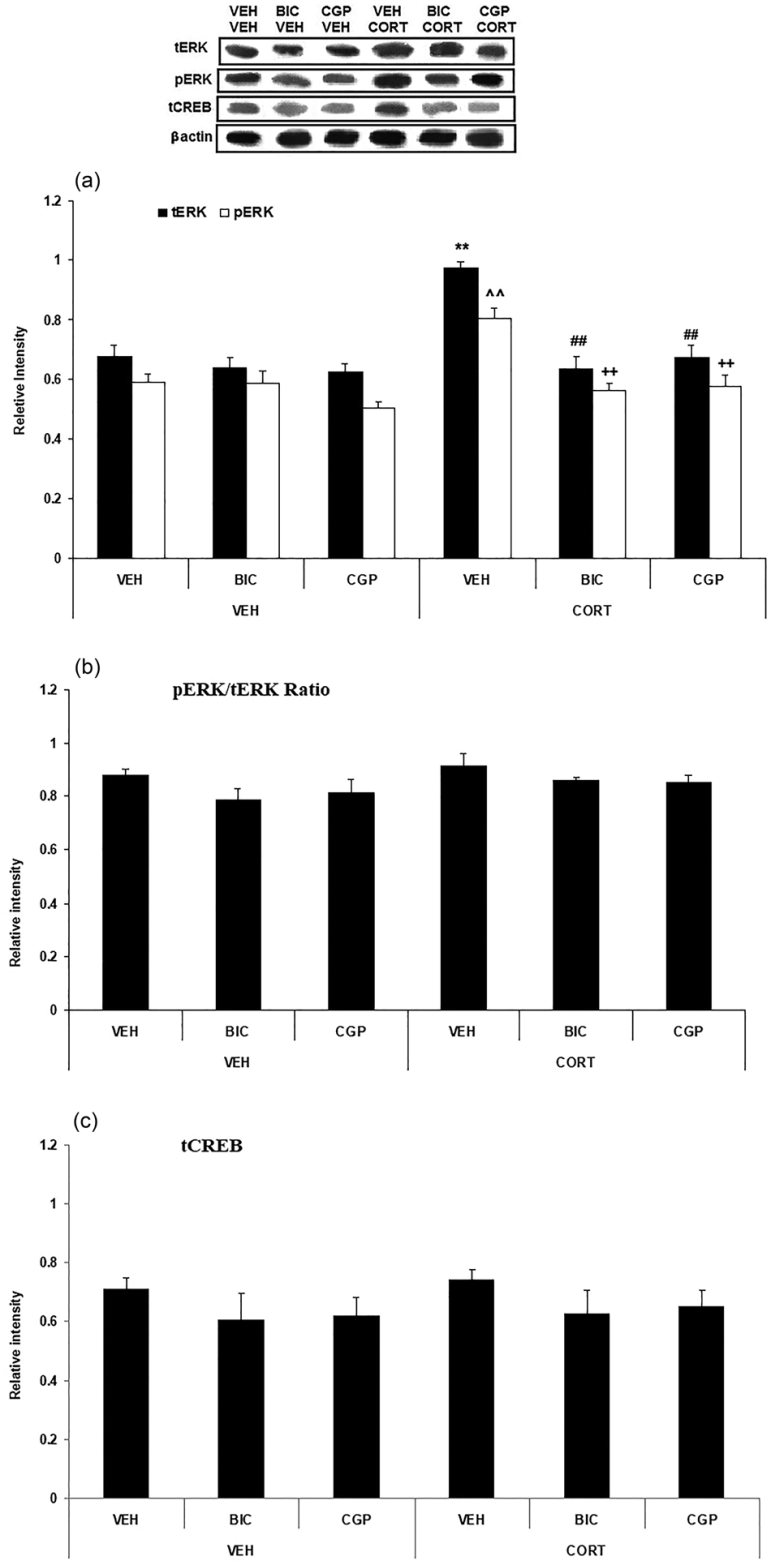
The effects of systemic corticosterone (CORT) injection on the levels of extracellular signal‐regulated kinase (ERK)1 and cAMP response element‐binding (CREB) within the infralimbic (IL) region following the intra‐IL administration of GABA_A_ or GABA_B_ receptor antagonists (bicuculine [BIC] or CGP55845 [CGP], respectively). (a) Total and phosphorylated levels of ERK1; (b) phosphorylated ERK1 (pERK1)/total ERK1 (tERK1) ratio; (c) CREB levels. Data represent the mean ± SEM. **p < .01 versus the tERK level in the vehicle (VEH)–VEH (control) group; ^^*p *< .01 versus the pERK1 level in the VEH–VEH (control) group; ##*p *< .01 versus the tERK1 level in the VEH–CORT group; ++*p *< .01 versus the pERK1 level in the VEH–CORT group. The data are presented as mean ± SEM. GABA, gamma‐aminobutyric acid.

This experiment investigated the effects of intra‐IL injection of GABA_A_, or GABA_B_ receptor antagonists, and systemic CORT administration on ERK1 and CREB levels in the IL. The data of ERK1 and CREB analysis are shown in Figure 6a–c.

A two‐way ANOVA analysis (CORT × GABA antagonist) on tERK1 relative intensity indicated significant effects of CORT (*F*_1, 18_ = 16.22, *p* = .0008) and GABA antagonist (*F*_2, 18_ = 18.41, *p* < .0001) and a significant interaction between two factors (*F*_2, 18_ = 10.65, *p* = .0009). Tukey's post hoc test revealed a significant elevation of tERK within the VEH–CORT 3 mg compared to the VEH–VEH group (*p* < .01). Furthermore, there was a significant reduction of tERK1 within the BIC 100 ng–CORT 3 mg and CGP 10 ng–CORT 3 mg compared to the VEH–CORT 3 mg group (*p* < .01) (Figure 6a).

A two‐way ANOVA analysis (CORT × GABA antagonist) on pERK1 relative intensity indicated significant effects of CORT (*F*_1, 18_ = 18.28, *p* = .0005) and GABA antagonist (*F*_2, 18_ = 19.84, *p* < .0001) and a significant interaction between two factors (*F*_2, 18_ = 6.38, *p* = .008). Tukey's post hoc test revealed a significant elevation of pERK1 within the VEH–CORT 3 mg compared to the VEH–VEH group (*p* < .01). Furthermore, there was a significant reduction of pERK1 within the BIC 100 ng–CORT 3 mg and CGP 10 ng–CORT 3 mg compared to the VEH–CORT 3 mg group (*p* < .01) (Figure 6a).

A two‐way ANOVA analysis (CORT × GABA antagonist) on pERK1/tERK1 ratio indicated no significant effects of CORT (*F*_1, 18_ = 2.77, *p* = .11) and GABA antagonist (*F*_2, 18_ = 2.62, *p* = .10) and no significant interaction between two factors (*F*_2, 18_ = 0.16, *p* = .85) (Figure 6b).

A two‐way ANOVA analysis (CORT × GABA antagonist) on tCREB relative intensity indicated no significant effects of CORT (*F*_1, 18_ = 0.27, *p* = .60) and GABA antagonist (*F*_2, 18_ = 1.76, *p* = .19) and no significant interaction between two factors (*F*_2, 18_ = .006, *p* = .99) (Figure 6c).

In summary, CORT substantially elevates the tERK1 and pERK1 within the IL region while not affecting tCREB levels. Moreover, the presence of BIC and CGP35348 distinctly suppressed the effects of CORT. These findings suggest that glucocorticoids and the GABAergic system may regulate fear extinction through the ERK pathway in the IL.

## 4 DISCUSSION

The key findings of the present study are as follows: (1) Systemic injection of CORT before the extinction test significantly potentiated fear memory extinction; (2) both systemic and intra‐IL injections of BIC and CGP55845 (GABA_A_ and GABA_B_ receptor antagonists, respectively) before CORT injection significantly inhibited the effects of CORT on fear memory extinction; (3) CORT significantly raised the tERK1 and pERK1 levels without eliciting a similar effect on CREB; and (4) the antagonists of GABA_A_ and GABA_B_ receptors impeded the impact of CORT on tERK1 and pERK1. These findings suggest that glucocorticoids and the GABAergic system may modulate fear extinction through the ERK pathway in the IL.

Although the focus was on elucidating the role of the IL region in the interactive effects of glucocorticoids and GABA receptors on fear extinction, we also recognized the importance of investigating these processes at a broader systemic level. The rationale for including systemic injections of the GABA_A_ receptor antagonist BIC and the GABA_B_ receptor antagonist CGP55845 was twofold: First, it allowed us to explore the global impact of GABAergic modulation on fear extinction, as multiple brain regions beyond the IL, such as the amygdala, hippocampus, and other prefrontal areas, are involved in this process. Second, replicating the findings from the intra‐IL injections through systemic administration strengthened the overall conclusions by demonstrating the robustness of the observed interactions between the glucocorticoid and GABAergic systems on fear extinction. The convergence of results from both systemic and targeted intra‐IL manipulations highlights the broad significance of our findings and their potential implications for understanding the neurobiology of fear regulation and developing novel therapeutic strategies for anxiety disorders.

On the other hand, the observation that the VEH–CORT group exhibited significantly lower freezing behavior in the first and second blocks of Ext 1 raises important considerations about the role of CORT in the retrieval of conditioned fear memory. Specifically, the reduced freezing levels observed during extinction trials suggest that CORT injection may impair the ability to recall fear memories, leading to a lasting decrease in freezing behavior across subsequent extinction sessions. Previous research has demonstrated that glucocorticoids, such as CORT, can modulate memory processes, including the retrieval of fear memories (de Quervain et al., 2017). For instance, Atsak et al. (2012) reported that glucocorticoid administration can impair the retrieval of contextual fear memories, potentially by interfering with neural circuits involved in memory recall. This aligns with our findings, where CORT administration appears to have a persistent effect on freezing behavior, suggesting a disruption in fear memory retrieval mechanisms.

### 4.1 The effects of GABA_A_ and GABA_B_ receptors inhibition on facilitatory effects of CORT on fear extinction

Glucocorticoids, natural hormones released from the adrenal cortex, have the ability to influence various types of learning and memory, including auditory and contextual fear memory. They affect different brain regions, such as the hippocampus, basolateral amygdala (BLA), and mPFC (Dadkhah et al., 2018). For instance, administering glucocorticoid agonists systemically or directly into the amygdala before extinction training has been shown to enhance the process of fear extinction in a manner dependent on the dosage. Conversely, blocking GRs in the amygdala with a substance, like RU38486, has been found to impede the extinction of conditioned fear (Yang et al., 2006). These findings underscore the significance of GRs in modulating fear memory. Additionally, studies have revealed that prolonged exposure to CORT before fear extinction can impair the extinction process without affecting fear memory recall (Gourley et al., 2009). Glucocorticoids, naturally released during stressful or emotionally charged events, aid fear extinction by reducing negative memory retrieval and enhancing extinction memory consolidation (Dadkhah et al., 2018; de Quervain et al., 2019; Omoumi et al., 2023). This property has been utilized to enhance exposure therapy for anxiety disorders. Consistent with these findings, our study showed that CORT facilitates the extinction of auditory‐conditioned fear memory.

We also found that intra‐IL injection of a GABA_A_ receptor antagonist (BIC) before CORT injection significantly inhibited the facilitative effects of CORT on fear memory extinction. It suggests that glucocorticoids may play a role in facilitating the extinction of fear memory via GABA_A_ neurotransmission. Akirav et al. (2006) have shown that injection of a low dose of muscimol (a GABA_A_ agonist) into the IL before fear extinction learning facilitates long‐term fear extinction. However, such an effect is not seen after the fear extinction learning. In addition, they found that intra‐BLA injection of muscimol after a brief extinction session enhances extinction memory up to 48 h after the drug was injected. They concluded that GABA_A_ neurotransmission plays a vital role in the onset and maintenance of fear extinction, with facilitating roles in both the IL and BLA. The GABA_A_ activation facilitates initiating and retaining fear extinction in the IL while facilitating the consolidation of extinction in the BLA. Reportedly, blockades of the GABAergic system in BLA, mPFC, and the hippocampus also impair extinction learning, and defects in the GABAergic system can lead to impaired fear extinction (Omoumi et al., 2023).

In addition, our study revealed that the facilitative effects of CORT on fear memory extinction were considerably impeded by intra‐IL injection of a GABA_B_ receptor antagonist (CGP35348) before CORT injection. GABA_B_ is a critical receptor for GABA neurotransmitters, primarily known for inhibitory function. The activation of GABA_B_ receptors hinders the release of neurotransmitters in presynaptic sites and hyperpolarizes the membrane potential in postsynaptic sites (Gassmann & Bettler, 2012; Newberry & Nicoll, 1984). Aberrant signaling in GABA_B_ receptors has been linked to anxiety and fear disorders (Cryan & Kaupmann, 2005), and clinical trials have utilized GABA_B_ agonists such as baclofen to treat PTSD (Manteghi et al., 2014). Furthermore, studies by Zhang et al. (2016) have demonstrated that GABA_B_ receptors play a role in fear extinction by influencing cholinergic neurons in the Habenula, resulting in decreased fear memory. The selective deactivation of GABA_B_ receptors disrupts the extinction of fear memory, whereas their activation reduces the expression of conditioned fear.

Previously, it was thought that GABA_B_ activity solely led to inhibitory responses. However, recent research has established that GABA_B_ is also involved in presynaptic excitation. Baclofen, which activates GABAB receptors, significantly enhances glutamatergic excitatory postsynaptic currents (Zhang et al., 2016). GABA_B_ may amplify the corelease of various neurotransmitters and potentiate certain neural circuits, such as the habenulo–interpeduncular pathway (Zhang et al., 2016). Given the dense distribution of GABAergic neurons in the mPFC (Bowery et al., 1987), baclofen reduces fear memory expression by enhancing neurotransmitter release. This is supported by findings showing that pharmacological activation of glutamate receptors and acetylcholine facilitates fear extinction (Zhang et al., 2016). Upon ligand binding to GABA_B_ receptors, the high‐voltage activated calcium channel Ca_v2.3_ opens, increasing calcium flow. This, in turn, releases glutamate, acetylcholine, and neurokinin B to activate interstitial neurons. The resulting increase in these neurotransmitters is associated with reduced fear responses (Zhang et al., 2016).

It is worth noting that clinical research has indicated a reduction in GABA levels in the brain regions that are involved in fear expression among patients with PTSD (Akirav & Maroun, 2007). This study highlights the joint influence of glucocorticoids and the GABAergic system in modulating the extinction of conditioned fear memory. Specifically, the GABA receptors in the IL region play a crucial role in regulating the effects of glucocorticoids on fear extinction, which might be leveraged to treat PTSD and its related complications by accelerating the process of fear memory extinction in these patients. Impairment of fear extinction may be crucial in stress and trauma‐related disorders, particularly PTSD. Numerous studies have linked alterations in the GABAergic system, which is the major neuroinhibitory system, to the pathophysiology of PTSD (Huang et al., 2023). Changes in GABA activity in different brain regions may be involved in fear extinction impairment. The inhibitory function of the GABAergic system may decrease or increase in various parts of the brain. Depending on the role of these structures in the formation and extinction of fear expression, GABA neurotransmission alterations could lead to disrupted fear extinction. For example, decreased GABA activity in the amygdala and increased GABA activity in the prefrontal cortex have been associated with impaired fear extinction and PTSD (Huang et al., 2023). Therefore, it is not surprising that the GABA receptors in the IL, a subregion of the brain that plays a significant role in fear extinction, would be involved in this process.

However, the present study found no specific effects on fear extinction when the GABA_A_ and GABA_B_ antagonists were administered alone before fear extinction. This could be due to various factors, such as the dose of the antagonists, the timing of injections, the task employed, and other issues that require further investigation. Nonetheless, GABA_A_ and GABA_B_ antagonists were found to inhibit the facilitative effects of CORT, indicating that fear extinction is jointly facilitated by the glucocorticoid and GABAergic systems. From a clinical perspective, these findings underscore the importance of the interaction between glucocorticoids and GABA systems in enhancing the elimination of pathological fear memory.

In general, fear extinction is significantly modulated by glucocorticoids and GABA receptors. Glucocorticoids, such as CORT, have the ability to influence synaptic plasticity by interacting with membrane‐bound receptors that mediate the effects of neurotransmitters, including GABA (Boguszewski, 2013). Previous research conducted by our team has demonstrated that glucocorticoids enhance fear memory extinction, which involves GABA receptors and the ERK pathway (Omoumi et al., 2023). Moreover, the endocannabinoid system, regulated by glucocorticoids, also contributes to the fear extinction. Within the mPFC, glucocorticoid signaling can activate endocannabinoid signaling, leading to decreased GABA release in the mPFC, thereby likely increasing the activity of principal neurons in the prelimbic region (Hill et al., 2011). GABA receptors, particularly GABA_A_ receptors, play a crucial role in fear extinction learning. GluN2D NMDA receptors, involved in gating fear extinction learning and interneuron plasticity, also influence GABA release from molecular layer interneurons (Dubois & Liu, 2021). Additionally, the diversity of GABA_A_ receptor subunits in major peripheral organs, as well as their plasticity in response to early‐life psychosocial stress, can affect fear extinction processes (Everington et al., 2018).

### 4.2 Role of ERK1 and CREB in fear memory extinction

ERK, a member of the MAPKs family, plays a crucial role in many cellular functions, including synaptic plasticity as well as learning and memory processes (Kelly et al., 2003). Signals that enter the cell are gathered and then conveyed to the nucleus by ERK. ERK is instrumental in determining the fate of fear memory and affects the shift of fear memory phases from reconsolidation to extinction (Merlo et al., 2018). Although reconsolidation and extinction utilize similar intracellular pathways, the underlying transcriptional mechanisms appear to be distinct (Mamiya et al., 2009). CREB, which is also involved in learning and memory processes, is a target of ERK (Lonze & Ginty, 2002).

In addition to their nongenomic effects, glucocorticoids are steroid hormones that act upon nuclear GRs. Consequently, ERK signaling contributes to at least a portion of the impact of glucocorticoids on fear extinction. Liu et al. (2015) conducted a study to determine the cellular and molecular mechanisms underlying the effect of novelty on extinction memory in rats. Using a contextual fear‐conditioning task, it was found that exposure to a novel environment enhanced long‐term extinction memory and reduced fear reinstatement. Further investigation revealed that novelty promoted extinction memory through hippocampal GR‐dependent and β‐adrenoceptor pathways and activated the Erk1/2‐CREB pathway, which acted as a behavioral tag of extinction. According to their suggestion, the critical roles in this process are played by hippocampal GRs and Erk1/2, but not β‐adrenoceptors (Liu et al., 2015). Our results demonstrated systemic glucocorticoid ligand (CORT) administration reduces fear expression during extinction sessions. Injection of CORT before the first extinction trial resulted in decreased fear memory and increased fear extinction. At the same time, CORT enhanced tERK1 and pERK1 but not CREB in the IL region. Systemic and intra‐IL administration of GABA_A_ and GABA_B_ receptor antagonists inhibited the enhancing effect of CORT on ERK1 activity. Based on this evidence, it can be concluded that the joint effects of glucocorticoids and the GABAergic system on fear extinction may be mediated through the ERK pathway. This finding is consistent with our recent study, which examined the interactive effect of glucocorticoids and β‐adrenoceptors in the IL region on the acquisition and consolidation of fear extinction in rats (Meamar, Rashidy‐Pour, Rahmani, et al., 2023).

The results showed that GRs and β‐adrenoceptors in the IL region collaborate to regulate fear memory extinction through the ERK1 and CREB signaling pathways. However, the results of this study did not demonstrate an enhancement of CREB activity. As mentioned earlier, CREB operates as a target and effector of ERK (Lonze & Ginty, 2002). Over time, ERK activation increases CREB activity. The CREB levels not rising in this experiment may be due to the timing of the CREB measurement. Further studies involving ERK and MAPK/ERK kinase inhibitors may help to clarify this issue.

One potential limitation of the present study is that only male rats were used as subjects. Evidence suggests sex differences in fear learning, memory consolidation, and extinction processes. For example, a recent study has reported that female rats show higher startle overall during fear‐potentiated startle recall and higher contextual fear than males (Olivera‐Pasilio & Dabrowska, 2023). Conversely, male rats tend to display enhanced fear extinction compared to females. These sex differences have been attributed to factors such as fluctuations in ovarian hormones, organizational effects of gonadal hormones during development, and sex‐specific neurobiology of fear circuits. Our findings may not fully capture the potential sex‐specific mechanisms underlying the interactions among the glucocorticoid system, GABAergic neurotransmission, and fear extinction by focusing exclusively on male subjects. Future studies should aim to investigate whether the observed effects of CORT and GABA receptor modulation on fear extinction and ERK signaling in the IL are consistent across both sexes or exhibit divergent patterns.

Another limitation of our study is that we did not assess the levels of phosphorylated CREB in the IL region. Although CREB is a well‐known downstream target of the ERK signaling pathway, we encountered technical difficulties in reliably measuring pCREB levels using our experimental setup. We acknowledge that examining pCREB could have provided additional insights into the molecular mechanisms underlying the interactive effects of glucocorticoids and the GABAergic system on fear extinction. However, due to the technical challenges faced, we could not include pCREB data in the present study. The last limitation of our study is the inability to assess ERK2 alongside ERK1 due to technical issues. Specifically, we encountered difficulties with the specificity and sensitivity of the antibodies for ERK2 in our Western blot analysis, which prevented us from obtaining reliable and interpretable data for this isoform. This limitation restricts our analysis to ERK1 and may overlook potential contributions of ERK2 in the observed molecular and behavioral effects. Future studies should aim to include both ERK1 and ERK2 to provide a more comprehensive understanding of the roles these isoforms play in fear extinction processes.

## 5 CONCLUSION

The injection of a systemic dose of CORT inhibited fear memory and enhanced fear extinction and phosphorylated and total forms of ERK1 in the IL. Systemic and intra‐IL injection of GABA_A_ and GABA_B_ receptor antagonists 15 min before CORT injection inhibited the facilitating effects of CORT on fear memory extinction and decreased ERK1 activity in the IL. These findings suggest that the glucocorticoid and GABAergic systems in the IL jointly facilitate fear extinction by affecting the ERK pathway. From a clinical point of view, our results may highlight the importance of the glucocorticoid‐GABA interaction in improving the extinction of pathological fear memory.

## AUTHOR CONTRIBUTIONS

**Samira Omoumi**; **Seyed Ali Seyedinia**; **Parnia Tarahomi**; and **Katayoun Sedaghat**: Investigation; methodology. **Ali Rashidy‐Pour**: Investigation; conceptualization; writing—review and editing. **Abbas Ali Vafaei**: Conceptualization; investigation; methodology; writing—review and editing; writing—original draft; supervision. **Payman Raise‐Abdullahi**: Writing—original draft; writing—review and editing; formal analysis.

## CONFLICT OF INTEREST STATEMENT

The authors declare no conflicts of interest.

### PEER REVIEW

The peer review history for this article is available at https://publons.com/publon/10.1002/brb3.70043

## Data Availability

The data that support the findings of this study are available from the corresponding author upon reasonable request.
